# MiR‐338 regulates NFATc1 expression and inhibits the proliferation and epithelial‐mesenchymal transition of human non‐small‐cell lung cancer cells

**DOI:** 10.1002/mgg3.1091

**Published:** 2019-12-11

**Authors:** Wei He, Jibin Lu

**Affiliations:** ^1^ Second Thoracic Surgery Ward Shengjing Hospital Affiliated to China Medical University Liaoning People’s Republic of China; ^2^ First Thoracic Surgery Ward Shengjing Hospital Affiliated to China Medical University Liaoning People’s Republic of China

**Keywords:** EMT, microRNA‐338, NFATc1, NSCLC, proliferation

## Abstract

**Background:**

It is well known that nuclear factor of activated T cells c1 (NFATc1) expression is closely associated with progression of many cancers. And we found that miR‐338 could directly target the NFATc1. However, the precise mechanisms of miR‐338 in non‐small‐cell lung cancer (NSCLC) have not been well clarified. Our study aimed to explore the interaction between NFATc1 and miR‐338 in NSCLC.

**Methods:**

Quantitative RT‐PCR was utilized to determine the expressions of NFATc1 and miR‐338 in NSCLC tissues and cell lines. And the cell proliferation and epithelial‐mesenchymal transition (EMT) were assessed to determine the functional roles of miR‐338 and NFATc1 in NSCLC cells. NFATc1 expression was detected using quantitative RT‐PCR and western blotting, respectively. Luciferase reporter assays were performed to validate NFATc1 as a target of miR‐338 in NSCLC cells.

**Results:**

In this study, our results showed that NFATc1 expression was significantly up‐regulated in NSCLC tissues and cell lines, and the miR‐338 level was dramatically down‐regulated. Moreover high NFATc1 expression was closely associated with low miR‐338 level in NSCLC tissues. Moreover introduction of miR‐338 significantly inhibited proliferation and EMT of NSCLC cells. Bioinformatics analysis predicted that the NFATc1 was a potential target gene of miR‐338. We demonstrated that miR‐338 could directly target NFATc1 by using luciferase reporter assay. Besides, knockdown of NFATc1 had the similar effects with miR‐338 overexpression on NSCLC cells. Up‐regulation of NFATc1 in NSCLC cells partially abolished the inhibitory effects of miR‐338 mimic.

**Conclusions:**

Overexpression of miR‐338 inhibited cell proliferation and EMT of NSCLC cells by directly down‐regulating NFATc1 expression.

## Graphical abstract

Overexpression of miR‐338 significantly inhibited the proliferation and epithelial‐mesenchymal transition of non‐small‐cell lung cancer (NSCLC) cell. Moreover introduction of nuclear factor of activated T cells c1 (NFATc1) reversed the inhibitory effects of miR‐338 overexpression. Therefore, our outcomes showed critical roles for miR‐338/ NFATc1 axis in the pathogenesis of NSCLC and suggested its possible application in tumor treatment.

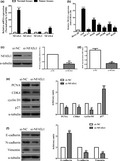

## 1 INTRODUCTION

Lung cancer, one of the most frequently diagnosed cancers, leads to the largest number of cancer‐related deaths worldwide (Zhang, Shao, Lin, Liu, & Yang, 2017; Zhang, Ding, & Zhang, 2017). According to the recent data, we know that the 5‐year survival rate of patients with lung cancer is only 15%. In all lung cancers, non‐small cell lung cancer (NSCLC) is the most common, which accounts for 85% of all lung cancers (Laskin & Sandler, 2009). Compared with the small cell lung cancer, it is difficult for NSCLC to be diagnosed due to relatively slow growth rate and late early metastasis (Peters, Zimmermann, & Adjei, 2014). When lung cancer is diagnosed, most of patients have advanced stage tumors (Jiang & Zhou, 2014). Therefore, it is an emergency requirement to find the available biomarkers and therapeutic strategies for the treatment of NSCLC.

The Nuclear factor of activated T‐cells (NFAT) family consists of five members: NFATc1 (also known as NFAT2), NFATc2 (NFAT1), NFATc3 (NFAT4), NFATc4 (NFAT3), and NFAT5 (Pan, Xiong, & Chen, 2013). More and more reports suggested that NFAT signaling plays a critical role in tumor growth and tumorigenesis (Jauliac et al., 2002). Particularly, isoform‐specific functions of NFAT in different types of neoplasms have been indicated in immune cells. Among these five isoforms, high expression of NFATc1 has often been determined in many types of cancers such as hepatic, pulmonary and pancreatic carcinomas, compared with their corresponding benign tissues (Buchholz et al., 2006; Chen et al., 2011; Wang et al., 2012). However, the expression and its role of NFATc1 in NSCLC are still unclear. Therefore, the aim of this study was to investigate the critical role of NFATc1 in NSCLC.

It is known that microRNAs (miRNAs) are participated in multiple biological processes including cell growth, migration, apoptosis, differentiation, inflammation, development, and stress response (Feng et al., 2018; Liu et al., 2015; Rothschild et al., 2012; Song et al., 2014; Wan, Zhang, Fan, & Wang, 2014). During past decades, a number of reports showed that miRNAs are either down‐regulated or up‐regulated, and play an important role in the carcinogenesis such as gastric cancer, gallbladder carcinoma, hepatocellular carcinoma, colorectal cancer, glioma, and bladder cancer (Feng et al., 2018; Garzon, Calin, & Croce, 2009; Li et al., 2015; Wang et al., 2017; Zhang, Shao, et al., 2017; Zhang, Ding, et al., 2017). Hence, studies of miRNAs in NSCLC are very critical to identify new diagnostic biomarker for lung cancer diagnosis.

In this study, we also found significant up‐regulation of NFATc1 in NSCLC tissues and cells. Knockdown of NFATc1 inhibited the proliferation and epithelial‐mesenchymal transition (EMT) of NSCLC cells. Up to now, the relationship between NFATc1 and miRNA processing in tumor progression remains little known. Then, we found that miR‐338 directly targeted NFATc1 in NSCLC. Overexpression of miR‐338 significantly inhibited the proliferation and EMT of NSCLC cell. Moreover introduction of NFATc1 reversed the inhibitory effects of miR‐338 overexpression. Therefore, our outcomes showed critical roles for miR‐338/ NFATc1 axis in the pathogenesis of NSCLC and suggested its possible application in tumor treatment.

## 2 MATERIALS AND METHODS

### 2.1 Patients and tissue samples

Twenty pairs of human NSCLC tissues and the adjacent normal tissues were collected in the Shengjing Hospital Affiliated to China Medical University from January 2016 to December 2018. The procedure was approved by the Ethical Committee of Shengjing Hospital Affiliated to China Medical University and written informed consent was obtained from all enrolled patients in this study. All the patients did not receive radiotherapy or chemotherapy or any other treatment before and after operation. The tissues were snap‐frozen in liquid nitrogen for the further analysis.

### 2.2 Cell culture and transient transfection

Human lung cancer cells A549, H1650, SPCA‐1, H460, SW900, H226, and H1299 and the bronchial epithelial cell line BEAS‐2B were cultured in RPMI 1640 (GIBCO) supplemented with 10% fetal bovine serum (GIBCO) and 1% penicillin/streptomycin at 37°C in a humidified incubator with 5% CO_2_.

The miR‐338 mimics, miR‐338 inhibitor, miR‐negative control (miR‐NC), anti‐miR‐NC, siRNA for NFATc1 (si‐NFATc1), and siRNA‐negative control (si‐NC) were synthesized and purified by Gene‐Pharma. The NFATc1‐overexpression plasmid was generated by inserting NFATc1 cDNA into a pcDNA3.1 vector. This plasmid was sequenced confirmed by Gene‐Pharma. miR‐338 mimics (50 nM), miR‐338 inhibitors (100 nM), si‐NFATc1 (100 nM) and NFATc1‐overexpression plasmid (100 nM) were transfected using Lipofectamine 2000 reagent (Thermo) following the manufacturer's protocols. Total RNA and protein were collected 48 hr after transfection.

### 2.3 RNA isolation and qRT‐PCR

Total RNA was isolated from cell lines and tissues using the Trizol according to the manufacturer's instructions. The RNA was reverse‐transcribed into cDNA using reverse transcription system (Thermo) with TaqMan Micro‐RNA Reverse Transcription Kit (Applied Biosystems) according to the manufacturer's protocols. The levels of miR‐143, miR‐124, miR‐338, miR‐137, and miR‐218 were detected by the ABI PRISM 7500 Sequence Detection System (ABI). U6 was taken as internal control. The expressions of NFATc1 also analyzed by SYBR Green and normalized to GAPDH. Primer sequences are showed in Table 1. Each experiment was in triplicate.

**Table 1:** Sequence of primers for qRT‐PCR

Gene	Primer sequence
NFATc1	F: 5'‐CACCGCATCACAGGGAAGAC‐3'
	R: 5'‐GCACAGTCAATGACGGCTC‐3'
NFATc2	F: 5'‐GATAGTGGGCAACACCAAAGTCC‐3'
	R: 5'‐TCTCGCCTTTCCGCAGCTCAAT‐3'
NFATc3	F: 5'‐AGACAGTCGCTACTGCAAGCCA‐3'
	R: 5'‐GCGGAGTTTCAAAATACCTGCAC‐3'
NFATc4	F: 5'‐GCACCGTATCACAGGCAAGATG‐3'
	R: 5'‐TCAGGATTCCCGCGCAGTCAAT‐3'
miR‐143	F: 5'‐GCAGTGCTGCATCTCTG‐3'
	R: 5'‐GAACATGTCTGCGTATCTC‐3'
miR‐124	F: 5'‐TTCACAGCGGACCTTGA‐3'
	R: 5'‐GAACATGTCTGCGTATCTC‐3'
miR‐338	F: 5'‐ATATCCTGGTGCTGAGTG‐3'
	R: 5'‐GAACATGTCTGCGTATCTC‐3'
miR‐137	F: 5'‐ATTGCTTAAGAATACGCGT‐3'
	R: 5'‐GAACATGTCTGCGTATCTC‐3'
miR‐218	F: 5'‐TGTGCTTGATCTAACCATG‐3'
	R: 5'‐GAACATGTCTGCGTATCTC‐3'
U6	F: 5'‐CTCGCTTCGGCAGCACA‐3'
	F: 5'‐AACGCTTCACGAATTTGCGT‐3'
GAPDH	F: 5'‐GAGTCAACGGATTTGGTCGTATTG‐3'
	R: 5'‐CCTGGAAGATGGTGATGGGATT‐3'

### 2.4 Cell proliferation assay

To study the role of miR‐338 mimic or inhibitor in proliferation of A549 cells, 5 × 10^3^ cells were seeded in 96‐well plate, and then incubated overnight in complete medium. After removing the medium, the cells were transfected with miR‐338 mimic or inhibitor for 48 hr at 37°C. Cell Proliferation ELISA‐BrdU (colorimetric) Kit (Roche Diagnostics) was used to detect the cells proliferation according to the manufacturer's protocols.

### 2.5 Protein extraction and western blot analysis

The cells were lysed in RIPA lysis buffer (Cell Signaling Technology Inc) containing protease inhibitor (Thermo). The protein concentration of each sample was measured using the BCA Protein Assay kit (Thermo). Equal quantities of protein samples were loaded on 10% SDS‐PAGE and transferred to PVDF membranes (Millipore). After blocking with 5% non‐fat milk, the membrane was incubated overnight at 4°C with primary antibodies such as NFATc1 (ab25916), PCNA (ab92552), CDK4 (ab108357), cyclin D1 (ab16663), p27 (ab32034), and alpha tubulin (ab52866) (Abcam); E‐cadherin (#14472), N‐cadherin (#13116), and Vimentin (#5741) (Cell Signaling Technology Inc). After washing and incubation with horseradish peroxidase‐conjugated antibody (Cell Signaling Technology Inc) for 2 hr at room temperature, blotted proteins were detected using an enhanced chemiluminescence system (Millipore) following the manufacturer's protocols.

### 2.6 Luciferase reporter assay

The wild‐type (WT) and mutant (MUT) 3'‐UTR of human NFATc1 were amplificated by PCR and cloned into the pGL3‐luciferase reporter plasmid (Promega). Cells were co‐transfected with WT or MUT pGL3‐NFATc1 vector, and miR‐338 mimics, inhibitor or negative control, using Lipofectamine 2000 (Thermo) for 48 hr. The both firefly and Renilla luciferase activity was measured using the commercial Dual‐Luciferase reporter assay system (Promega) according to the manufacturer's instructions.

### 2.7 Statistical analysis

The data were expressed as the mean ± standard error of the mean. The number of independent experiments was represented by “n.” Multiple comparisons were performed using one‐way ANOVA followed by Tukey's multiple‐comparison test. Other comparisons were analyzed using two‐tailed Student's *t* test. *p* < .05 was considered statistically significant differences.

## 3 RESULTS

### 3.1 High expression of NFATc1 was in NSCLC specimens and its effects on cell proliferation and EMT of NSCLC cells

**Figure 1:**
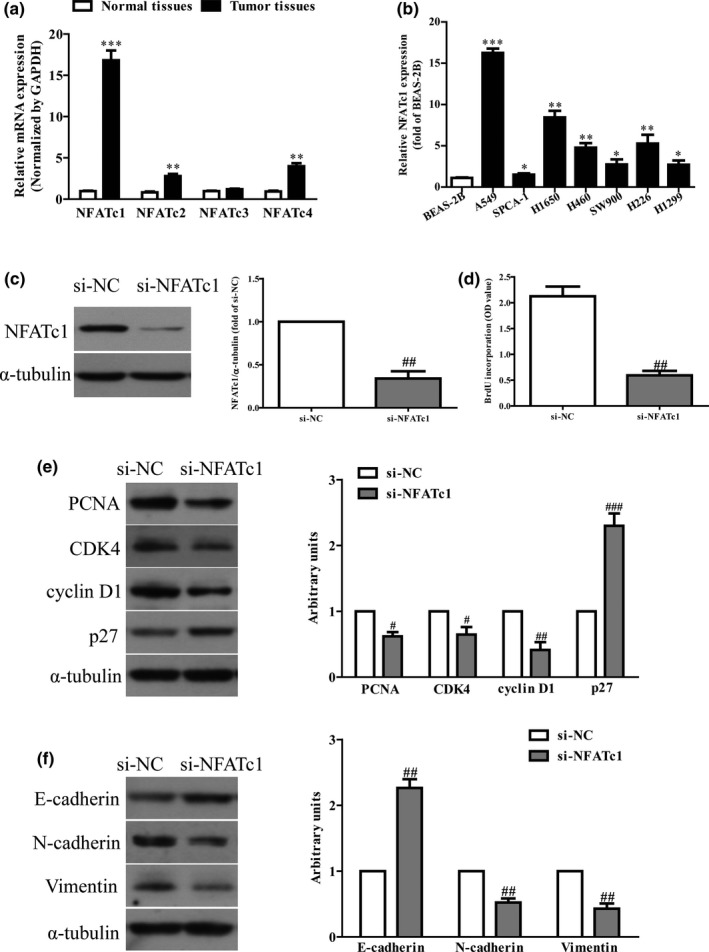
Expression and its effects of NFATc1 in NSCLC tissues and cell lines. (a) qRT‐PCR analysis of NFATc1, NFATc2, NFATc3, and NFATc4 expression in 20 pairs NSCLC tissues and the adjacent normal tissues. Transcript levels were normalized by GAPDH expression. (b) Relative NFATc1 expression analyzed by qRT‐PCR in seven NSCLC cell lines (A549, H1650, SPCA‐1, SW900, H460, H226, and H1299) and the bronchial epithelial cell line BEAS‐2B were normalized with GAPDH. A549 cells were transfected with si‐NFATc1 or si‐NC. (c) The protein expression of NFATc1 was determined by western blot. (d) Cell proliferation was assessed by Brdu assay. (e) The protein expressions of PCNA, CDK4, cyclin D1 and p27 were determined by western blot. (f) The expressions of E‐cadherin, Vimentin, and N‐cadherin were detected by western blot. All data are presented as mean ± *SEM*, *n* = 4. *p < .05, **p < .01, ***p < .001 versus. NSCLC tissues or BEAS‐2B; ^#^ *p* < .05, ^##^ *p* < .01, ^###^ *p* < .001 versus si‐NC. NFATc1, nuclear factor of activated T cells c1; NSCLC, non‐small‐cell lung cancer

It has been reported that NFAT family including NFATc1, NFATc2, NFATc3, and NFATc4 were closely associated with many kinds of cancers (Jauliac et al., 2002). Here, we tested these four NFAT genes in NSCLC tissues. Our findings indicated that the mRNA level of NFATc1 was the highest in NSCLC tissues among these four NFAT genes compared with the adjacent tissues (Figure 1a). To investigate the functional roles of NFATc1 in NSCLC, several NSCLC cell lines were determined. Subsequently, we also determined the level of NFATc1 in several NSCLC cell lines including A549, SPCA‐1, H1650, H460, SW900, H226, H1299 and a normal human bronchial epithelial cell line BEAS‐2B. Compared with BEAS‐2B, the level of NFATc1 in A549 cells was highest among these seven NSCLC cell lines (Figure 1b). We used A549 cells in the following experiments for further study, because its NFATc1 expression is exceptionally high.

Next, the proliferation and EMT of A549 cells were detected after transfection with si‐NC or si‐NFATc1. The NFATc1 expression was significantly decreased in A549 cells transfected with si‐NFATc1 compared with the si‐NC group (Figure 1c). The Brdu assay demonstrated that down‐regulation of NFATc1 could inhibit the proliferation of NSCLC cells (Figure 1d). Moreover western blot assay also confirmed that silencing NFATc1 significantly decreased the expressions of PCNA, CDK4, cyclin D1 and increased the expression of p27 at protein level (Figure 1e). Next, the EMT of NSCLC cells were suppressed after silencing NFATc1 expression, by enhancing E‐cadherin expression and reducing N‐cadherin and Vimentin expressions (Figure 1f). Altogether, these results demonstrated that NFATc1 was an oncogene in NSCLC.

### 3.2 miR‐338 directly targeted NFATc1 3'UTR

**Figure 2:**
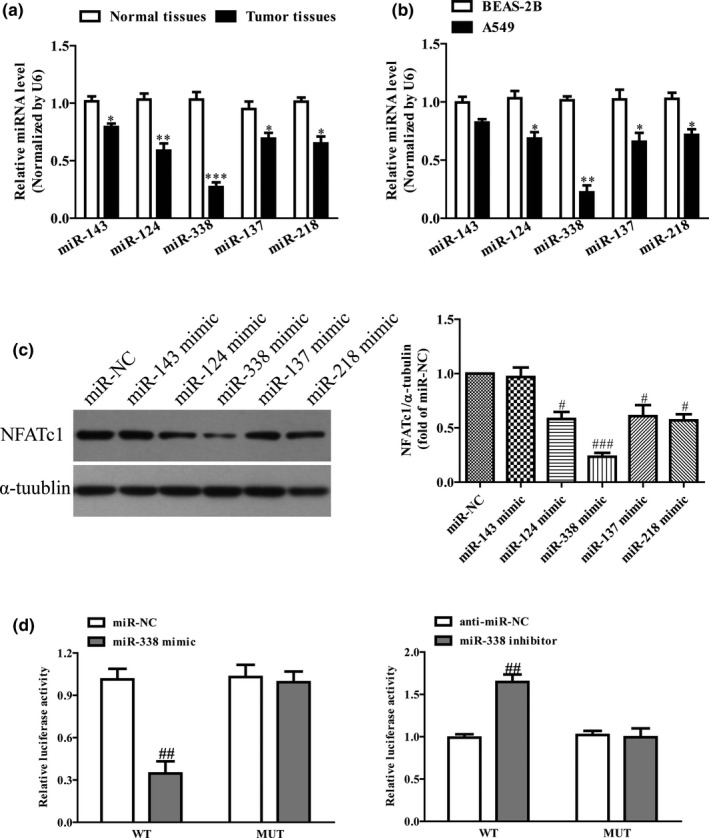
miR‐338directly targets NFATc1. (a) qRT‐PCR analysis of miR‐143, miR‐124, miR‐338, miR‐137, and miR‐218 levels in 10 pairs NSCLC tissues and the adjacent normal tissues. Transcript levels were normalized by U6 level. (b) qRT‐PCR analysis of miR‐143, miR‐124, miR‐338, miR‐137, and miR‐218 levels in A549 BEAS‐2B. (c) The protein expression of NFATc1 was determined by western blot. A549 cells were transfected with miR‐NC, miR‐143, miR‐124, miR‐338, miR‐137, and miR‐218 mimics for 48 hr, respectively. (d) The analysis of the relative luciferase activities of NFATc1‐WT, NFATc1‐MUT. All data are presented as mean ± *SEM*, *n* = 4. *p < .05, **p < .01, ***p < .001 versus. NSCLC tissues or BEAS‐2B; ^#^ *p* < .05, ^##^ *p* < .01, ^###^ *p* < .001 versus miR‐NC or anti‐miR‐NC. NFATc1, nuclear factor of activated T‐cells c1; NSCLC, non‐small‐cell lung cancer

To further study which miRNA regulated NFATc1 expression, we predicted several miRNAs including miR‐143, miR‐124, miR‐338, miR‐137, and miR‐218 by online database TargetScan 7.2, and these five miRNAs acted as tumor suppressor in lung cancer. Therefore, we determined the levels of miR‐143, miR‐124, miR‐137, miR‐218, and miR‐338 in NSCLC tissues and cell line. Our results showed that the miR‐338 levels were lowest among these five miRNAs in both NSCLC tissues (Figure 2a) and A549 cells (Figure 2b) compared with the adjacent tissues and BEAS‐2B. For further study, we found that the miR‐338 had stronger effect than other four miRNAs to down‐regulate the NFATc1 expression (Figure 2c). To further confirm NFATc1 as a miR‐338 target, our findings showed that miR‐338 significantly inhibited the luciferase activity of the 3'UTR NFATc1‐WT group compared with that in the NFATc1‐MUT group, whereas decreased miR‐338 level could enhance the luciferase activity of the 3'UTR NFATc1‐WT group (Figure 2d). Altogether, these results strongly supported that miR‐338 directly targeted NFATc1 in NSCLC cells.

### 3.3 Up‐regulation of miR‐338 inhibited of NSCLC cells proliferation

**Figure 3:**
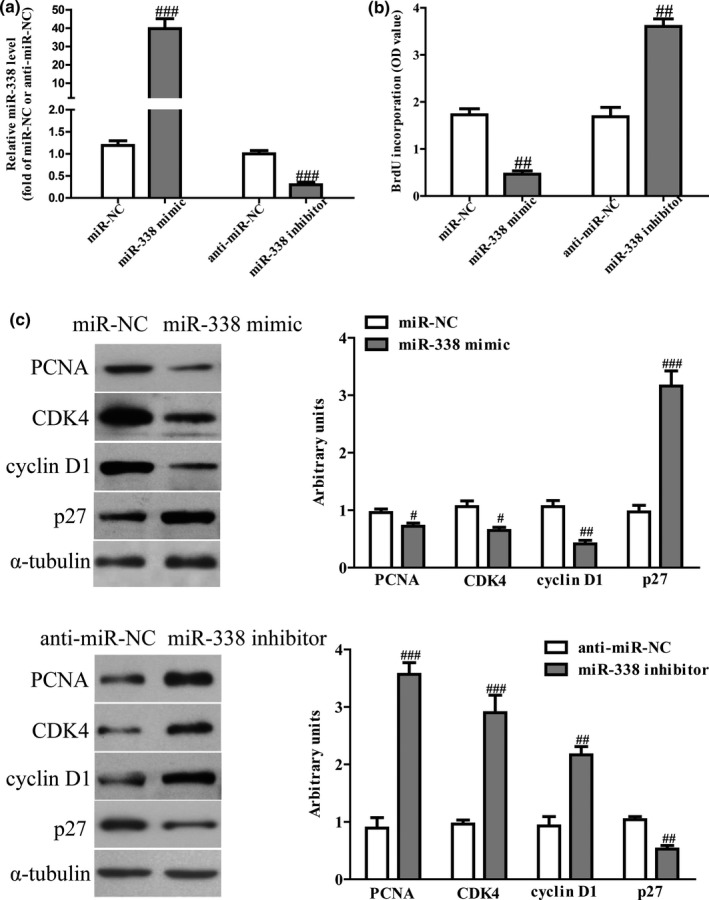
Effects of miR‐338 on the proliferation and related genes expressions in NSCLC cells. A549 cells were transfected with miR‐338 mimic or inhibitor for 48 hr. (a) The levels of miR‐338 in NSCLC cells were determined by qRT‐PCR. (b) Cell proliferation was assessed by Brdu assay. (c) The expressions of PCNA, CDK4, cyclin D1 and p27 were determined by western blot. All data are presented as mean ± *SEM*, *n* = 4. ^#^ *p* < .05, ^##^ *p* < .01, ^###^ *p* < .001 versus miR‐NC or anti‐miR‐NC. NSCLC, non‐small‐cell lung cancer

Next, we study the effects of miR‐338 on the proliferation of NSCLC cells. After transfection with miR‐338 mimic and inhibitor, the qRT‐PCR analysis showed that the level of miR‐338 was significantly up‐regulated in miR‐338 mimic group and down‐regulated in miR‐338 inhibitor group compared to NC group (Figure 3a). The results from CCK‐8 assay showed that increased level of miR‐338 markedly inhibited the proliferation of A549 cells (Figure 3b). To further confirm above results, we detected the effects of miR‐338 on expressions of several proliferation and cell cycle‐related molecules. Up‐regulation of miR‐338 decreased the expressions of PCNA, CDK4, cyclin D1 and increased the expression of p27 in NSCLC cells (Figure 3c). However, down‐regulation of miR‐338 significantly promoted NSCLC cells proliferation (Figure 3b), increased the expressions of PCNA, CDK4, cyclin D1 and decreased the expression of p27 in NSCLC cells (Figure 3c).

### 3.4 Overexpression of miR‐338 inhibited the EMT of NSCLC cells

**Figure 4:**
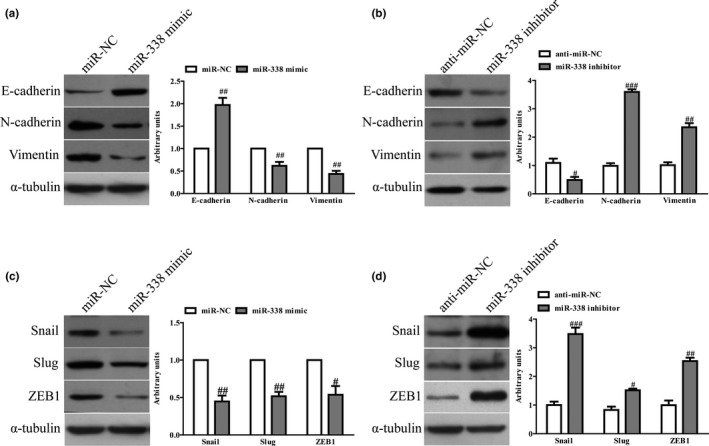
Effects of miR‐338 on EMT of NSCLC cells. A549 cells were transfected with miR‐338 mimic or inhibitor for 48 hr. (a, b) The expressions of E‐cadherin, Vimentin, and N‐cadherin were detected by western blot assays. (c, d) The expressions of Snail, Slug, and ZEB1 were detected by western blot assays. All data are presented as mean ± *SEM*, *n* = 4. ^#^ *p* < .05, ^##^ *p* < .01, ^###^ *p* < .001 versus miR‐NC or anti‐miR‐NC. EMT, epithelial‐mesenchymal transition; NSCLC, non‐small‐cell lung cancer

The EMT is one of the key initiation steps in the metastasis process. Hence, we explored whether miR‐338 regulated the EMT process to inhibit NSCLC progression. We found that miR‐338 overexpression lead to up‐regulation of the epithelial marker E‐cadherin. By contrast, the mesenchymal markers N‐cadherin and Vimentin were down‐regulated in miR‐338 overexpressed A549 cells (Figure 4a), while the miR‐338 inhibitors showed the opposite effect (Figure 4b). Furthermore, the expressions of EMT‐related transcription factors were also determined in A549 cells after transfection with miR‐338 mimic and inhibitor. Overexpression of miR‐338 significantly reduced the expressions of Snail, Slug, and ZEB1 in A549 cells (Figure 4c). However, knockdown of miR‐338 enhanced the expressions of these three EMT‐related transcription factors in NSCLC cells (Figure 4d). Thus, we demonstrated that miR‐338 could suppress the NSCLC progression by repressing cells proliferation and EMT.

### 3.5 Introduction of NFATc1 partially abolished the effects of miR‐338 mimic on proliferation and EMT of NSCLC cells

**Figure 5:**
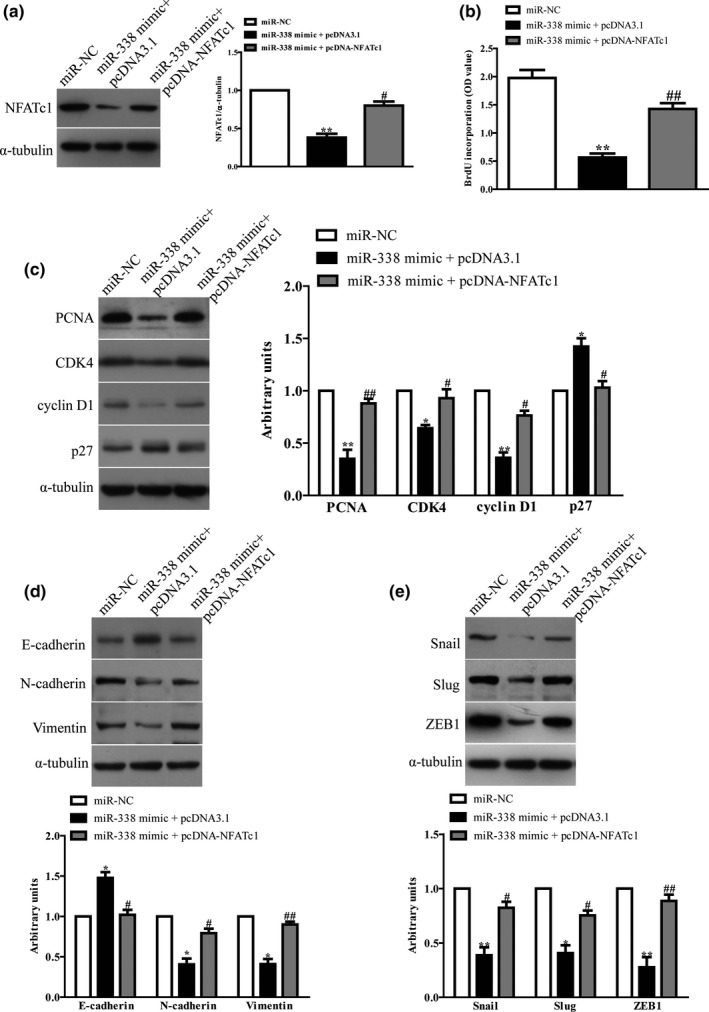
Overexpression of NFATc1 partially promoted cell proliferation and EMT in miR‐338‐overexpressing NSCLC cells. A549 cells were transfected with miR‐NC or miR‐338 mimic with or without pcDNA‐NFATc1 vector. (a) The protein expression of NFATc1 was determined by western blot. (b) Cell proliferation was assessed by Brdu assay. (c) The protein expressions of CDK4, cyclin D1 and p27 were determined by western blot. (d) The expressions of E‐cadherin, Vimentin and N‐cadherin were detected by western blot assays. (e) The expressions of Snail, Slug, and ZEB1 were detected by western blot assays. All data are presented as mean ± *SEM*, *n* = 4. *p < .05, **p < .01 versus. miR‐NC; ^#^ *p* < .05, ^##^ *p* < .01 versus. miR‐338 mimic. EMT, epithelial‐mesenchymal transition; NFATc1, nuclear factor of activated T‐cells c1; NSCLC, non‐small‐cell lung cancer

Next, we determined whether miR‐338 targeted NFATc1 to inhibit the proliferation and EMT of NSCLC cells. Then, we cotransfected pcDNA‐NFATc1 vector or its negative control (pcDNA3.1) with miR‐338 mimic or miR‐NC into A549 cells (Figure 5a). The data from cell proliferation assay suggested that concomitant overexpression of miR‐338 and NFATc1 abrogated the inhibitory effect of miR‐338 mimic (Figure 5b). Moreover, the expressions of PCNA, CDK4, cyclin D1 were up‐regulated and the p27 expression was down‐regulated in miR‐338‐overexpressing NSCLC cells after exogenous introduction of NFATc1 (Figure 5c). Next, increased NFATc1 expression could also reverse the inhibitory effect of the miR‐338 overexpression on EMT markers of NSCLC cells (Figure 5d), decrease the expression of E‐cadherin enhanced by miR‐338 mimic, and increase the expressions of N‐cadherin and Vimentin reduced by miR‐338 mimic. Moreover, miR‐338 mimic‐inhibited EMT‐related transcription factors of NSCLC cells were promoted by overexpression of NFATc1 (Figure 5e). Therefore, the inhibitory effects of miR‐338 were reversed by NFATc1 overexpression. Altogether, all above results convincingly demonstrated that miR‐338 inhibited cell proliferation and EMT in NSCLC cells by down‐regulation of NFATc1, and miR‐338 targeting NFATc1 was responsible for inhibition of the proliferation and EMT of NSCLC cells.

## 4 DISCUSSION

NSCLC is the most common lung cancer. Although the diagnosis and treatment of lung cancer continue to improve, the mortality rate remains high (Siegel, Miller, & Jemal, 2017). Therefore, it is of utmost importance to study the mechanism underlying the tumorigenesis and progression of lung cancer and identify gene targets for its diagnosis and treatment. The carcinogenic properties of NFATc1 have been further highlighted in recent studies. For example, Li, Duan, Yu, and Dang (2016) indicated that NFATc1 silencing regulates the cell cycle, apoptosis, invasion and migration of ovarian cancer cells. Liu et al. (2019) found that NFATc1 down‐regulation could suppress the proliferation, migration and invasion abilities of Prostate cancer cells, probably by regulating c‐myc and PKM2 expression. Wang, Wang, et al. (2015) and Wang, Xu, et al. (2015) identified that NFATc1 promoted invasion of human glioblastoma multiforme cells through the induction of cyclooxygenase‐2. However, the precise role of NFATc1 in malignant NSCLCs has not been elucidated. Our study found that NFATc1 mRNA level was up‐regulated significantly in NSCLC tissues and cell lines. Additionally, functional investigation revealed that siRNA‐mediated knockdown of NFATc1 led to a reduction of cell growth and EMT in NSCLC cells.

Cell cycle regulation involved complex events. Such events revealed that cell cycle related proteins provided a promising mechanism for the inhibition of growth (Qiu, Ma, Yang, Wang, & Jiang, 2017; Wang, Wang, et al., 2015; Wang, Xu, et al., 2015). An earlier study suggested that the up‐regulation of cyclin D1 and cyclin‐dependent kinase 2 were involved in cell cycle progression and arrested cells at the G0/G1 phase (Wang, Wang, et al., 2015; Wang, Xu, et al., 2015). The p27 has been reported as an inhibitor of CDK protein with an anti‐proliferative effect on mesangial cells (Wang et al., 2011). In our study, we found that knockdown of NFATc1 increased the expressions of cyclin D1 and CDK4 while it inhibited p27 level at protein level. These results meant that decreased NFATc1 expression inhibited the proliferation of NSCLC cells.

The EMT is a developmental process in which epithelial cells lose polarity and develop a mesenchymal phenotype (Chen et al., 2019). Increasing studies have demonstrated that aberrant activation of EMT leads to tumor invasion, metastatic dissemination and acquisition of therapeutic resistance (Radisky, 2005; Zavadil, Haley, Kalluri, Muthuswamy, & Thompson, 2008). At the molecular level, EMT is characterized by down‐regulation of epithelial marker E‐cadherin and cytokeratins, with up‐regulation of mesenchymal markers like N‐cadherin, Vimentin, and fibronectin (Gheldof & Berx, 2013; Yao, Dai, & Peng, 2011). Moreover, a number of transcription factors, such as Snail, Slug, Twist1, ZEB1, and ZEB2 are involved in mediating the phenotypic changes associated with EMT (Stemmler, Eccles, Brabletz, & Brabletz, 2019). It has been reported that NFATc1 is involved in regulation of EMT (Singh et al., 2015), which prompts us to investigate its roles in regulating EMT process. As expected, knockdown of NFATc1 enhanced epithelial marker E‐cadherin expression and reduced mesenchymal markers such as N‐cadherin and Vimentin expressions. This involvement of NFATc1 in regulating EMT provided an explanation for the higher NFATc1 expression in NSCLC tissues compared to the adjacent tissues.

In recent years, many studies have demonstrated that microRNAs are involved in the development and progression of lung cancer. Over the past decades, a number of reports have confirmed that miRNAs can act as a tumor regulator, either as cancer suppressor or oncogene (Barger & Nana‐Sinkam, 2015; Kang & Lee, 2014). It is well known that miRNAs perform their function by regulating the expression of its target gene. Previous studied have reported that miR‐338 can act as either oncogene or tumor suppressor (Chu et al., 2019; Zhang et al., 2019). For example, Zhang et al. (2019) found that miR‐338 suppressed the proliferation, invasion, and EMT of bladder cancer cells by decreasing ETS1. Chu et al. (2019) showed that miR‐338 promoted migration, invasion and EMT of colorectal cancer by regulating PIK3C3‐related autophagy pathway. In our study, the online database Targetscan 7.2 predicted that NFATc1 might be the functional target gene of miR‐338. The luciferase reporter assay demonstrated that miR‐338 could directly target NFATc1. Furthermore, overexpression or knockdown of miR‐338 significantly reduced or enhanced NFATc1 expression, respectively. The present study explored the expression of miR‐338 in the NSCLC. We demonstrated that the miR‐338 level was also down‐regulated in NSCLC tissues when compared with the adjacent normal tissues, which was consistent with previous results (Zhang, Shao, et al., 2017; Zhang, Ding, et al., 2017). In addition, the level of miR‐338 was also down‐regulated in NSCLC cell lines. For further study, we found that the miR‐338 level was inversely correlated with NFATc1 expression in NSCLC.

For further study the role of miR‐338 in NSCLC cells, the proliferation and EMT of NSCLC cells were detected after transfection with miR‐338 mimic or inhibitor. Increased expression of miR‐338 significantly suppressed NSCLC cell proliferation and EMT in vitro. However, knockdown of miR‐338 had the opposite effect on miR‐338 mimic. Besides, our results revealed that NFATc1 overexpression partially abolished the effects of miR‐338 mimic on the proliferation of NSCLC cells and reversed miR‐338 caused EMT‐related marker changes, indicating that miR‐338 could modulate NSCLC progression by directly targeting NFATc1.

In summary, our study suggested that miR‐338 could serve as a potential tumor suppressor in NSCLC progression. Furthermore, miR‐338 suppressed NSCLC cell proliferation and EMT by directly targeting NFATc1.

## CONFLICT OF INTEREST

The authors declare that they have no conflict of interest.

## AUTHORS CONTRIBUTION

In this work, WH and JL conceived the study and designed the experiments. WH and JL contributed to the data collection, performed the data analysis, and interpreted the results. WH wrote the manuscript. JL contributed to the critical revision of article. All authors read and approved the final manuscript.
